# Improvement of granulomatous skin conditions with tofacitinib in three patients: A case report

**DOI:** 10.1177/2050313X211039477

**Published:** 2021-08-17

**Authors:** Meghan L McPhie, William C Swales, Melinda J Gooderham

**Affiliations:** 1 Queen’s University, Kingston, ON, Canada; 2 SKiN Centre for Dermatology, Peterborough, ON, Canada; 3 Probity Medical Research, Waterloo, ON, Canada

**Keywords:** Granuloma annulare, necrobiosis lipoidica, tofacitinib, Janus kinase inhibitors, case report

## Abstract

Granulomatous skin conditions are poorly understood inflammatory skin diseases consisting predominantly of macrophages. Granuloma annulare (GA) is the most common granulomatous skin disease and the generalized variant is particularly difficult to treat due to the prolonged course and lack of efficacious treatment options. Necrobiosis lipoidica (NL) is another granulomatous disorder of uncertain etiology. There is a growing body of evidence for the use of Janus kinase (JAK) inhibitors in the management of inflammatory skin diseases. In our report, we describe three patients with recalcitrant granulomatous disease including NL and generalized GA who responded favourably to treatment with the JAK inhibitor tofacitinib. JAK inhibitors may be a beneficial therapeutic option for patients with granulomatous skin diseases that are unresponsive to conventional therapies. Further research is required to determine the long-term efficacy and safety of JAK inhibitors in treating granulomatous skin conditions.

## Introduction

Granulomatous skin conditions are poorly understood inflammatory skin diseases which predominantly involve the macrophage. Further complicating our understanding are the multiple morphologies and variants, including granuloma annulare (GA), necrobiosis lipoidica (NL), and sarcoidosis.^1,2^ GA is the most common granulomatous skin condition and is characterized by skin-coloured to red-brown papules and plaques often in an annular arrangement. The localized form of GA commonly presents on the hands or feet; it tends to be self-limited, with many cases spontaneously resolving within 2 years.^1^ Conversely, generalized GA is characterized by more widespread involvement of the trunk and extremities and can have a significant impact on patients’ quality of life.^3^ Unfortunately, generalized GA is more difficult to treat due to its protracted course and the paucity of efficacious, evidence-based therapeutic options.^4^ While no gold standard of care exists, management options for GA range from localized therapies such as topical and intralesional corticosteroids to systemic therapies, including phototherapy and systemic drug therapy such as isotretinoin, antimicrobials, and immunosuppressants.^4^

NL is a rare chronic granulomatous condition which is felt to be caused by microangiopathic changes leading to collagen degeneration. It differs in morphology from GA with a yellowish brown colour, central atrophy, and risk of ulceration.^5^ Causality of NL is uncertain; however, there is an association with diabetes, obesity, lipid disorders, and hypertension.^6^ Similar to generalized GA, NL is a difficult disease to manage, with conventional treatment options such as topical and intralesional corticosteroids, immunosuppressants, antiplatelet therapy, and phototherapy producing minimal and inconsistent results.^7^

The Janus Kinase/Signal Transducer and Activator of Transcription (JAK-STAT) pathway is an intracellular signalling network that mediates cellular responses involved in inflammation.^8^ Tofacitinib is a JAK inhibitor approved for use in four inflammatory conditions: rheumatoid arthritis (RA), psoriatic arthritis, juvenile idiopathic arthritis, and ulcerative colitis. JAK inhibitors are emerging as promising therapeutic agents in dermatology as well.^9^ While there is growing evidence for the efficacy of JAK inhibitors for treating inflammatory skin diseases such as atopic dermatitis, psoriasis, vitiligo, and alopecia,^9^ the use of JAK inhibitors for granulomatous conditions has been relatively unexplored beyond case reports.^10–17^ Increased knowledge of the role of JAK signalling in the inflammatory pathways involved has been reported.^18^

## Cases

We observed the outcomes of three patients with biopsy-proven granulomatous diseases (GA, NL) treated with tofacitinib between July 2020 and April 2021 in an outpatient dermatology clinic. Patient characteristics are presented in Table 1.

**Table 1.** Patient characteristics.

Patient	Age (yr)/sex	Comorbidities	Disease and duration, years	Prior therapies	Tofacitinib dose	Response to tofacitinib	Tofacitinib duration
1	71M	DM, DLD, HTN, arteriosclerosis, esophagitis, COPD, diabetic neuropathy	NL, 15y	TCS, TCI, ILK, cephalexin, prednisone, ustekinumab (Dec. 2018 – Jul. 2020), acitretin (Jul. 2019 – Nov. 2020), pentoxifylline (Dec. 2019 – Jan. 2021), HCQ (Apr. 2020 – Jan. 2021)	5 mg BID	Improved	10 months (ongoing)
2	78 F	RA, OA, Meniere’s disease	GA, > 6y	ILK, TCI, MTX (Jul. 2016 – Jan. 2019), phototherapy, ustekinumab (Jan. 2020 – Jul. 2020)	5 mg BID	Almost clear	9 months (ongoing)
3	59 F	HTN	GA, 10y	ILK, TCS, TCI, phototherapy, ustekinumab (Dec. 2019- Jun. 2020)	5 mg BID	Improved	4 weeks (discontinued due to coverage issues)

DM: diabetes; DLD: dyslipidemia; HTN: hypertension; COPD: chronic obstructive pulmonary disease; NL: necrobiosis lipoidica; TCS: topical corticosteroids; TCI: topical calcineurin inhibitors; ILK: intralesional Kenalog; HCQ: hydroxychloroquine; BID: bis in die (twice daily); RA: rheumatoid arthritis; OA: osteoarthritic; GA: granuloma annulare; MTX: methotrexate.

**Figure 1.**
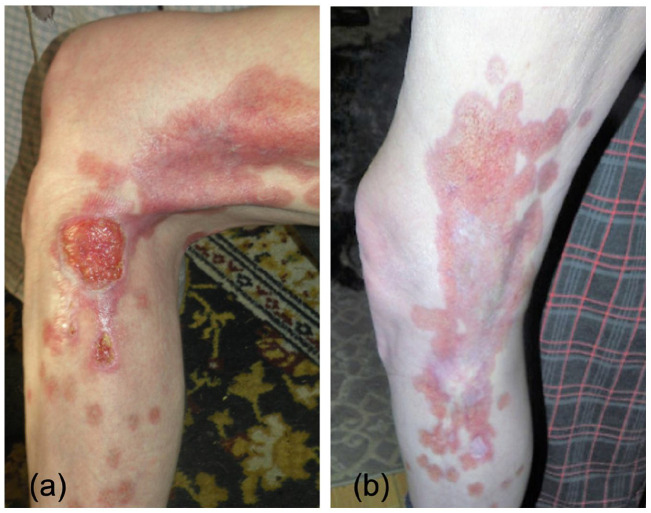
Effect of oral tofacitinib treatment for necrobiosis lipoidica. (a) Clinical image prior to oral tofacitinib therapy and (b) 6 months after oral tofacitinib therapy.

Patient 1 is a diabetic man in his 70s with a 15-year history of a progressive severe ulcerating granulomatous skin disease associated with debilitating lesional pain. For the first 9 years, he had asymptomatic localized disease, but subsequently developed widespread large erythematous annular plaques involving his neck, trunk, arms, and legs with areas of painful ulceration. Multiple skin biopsies were reported initially as GA and later as NL. The patient had undergone several failed therapies, including topical and intralesional corticosteroids, cephalexin, hydroxychloroquine, acitretin, pentoxifylline, and ustekinumab 90 mg q8 weeks, many of which were prescribed in combination. He was initiated on off-label tofacitinib 5 mg twice daily (BID) in July 2020. After 4 weeks, there was rapid marked improvement, with decreased erythema and flattening of the lesions. Ulcerated lesions on the leg completely healed and he had significant improvement in his pain (see Figure 1). After 10 months of follow-up, he continued to have improvement with no new lesions and no progression of existing lesions. He experienced no adverse effects during treatment.

Patient 2 is a 78-year-old female with RA and generalized GA, diagnosed over 6 years ago. She had widespread erythematous annular plaques involving her trunk, buttocks, and extremities with a significant impact on her quality of life. Previous treatments were not effective, including topical and intralesional corticosteroids, phototherapy, methotrexate, and ustekinumab 90 mg q8 weeks. She was started on tofacitinib 5 mg BID for both her GA and concomitant RA. At her 4-week follow-up, in addition to helping her RA, there was rapid improvement in the skin lesions. After 9 months of treatment, she is almost clear, but still has some residual annular areas on her left leg.

Patient 3 is a 59-year-old female with a 10-year history of generalized GA, characterized by erythematous annular plaques affecting her trunk, extremities, neck, and face. Previous treatment with topical and intralesional corticosteroids, topical calcineurin inhibitors, phototherapy, and ustekinumab 90 mg q8 weeks had failed. She was initiated on off-label tofacitinib 5 mg BID. She noticed rapid improvement after 1 week of treatment and after 4 weeks, she had thinning of the plaques and had not developed any new lesions. Unfortunately, the patient was unable to secure insurance coverage for tofacitinib beyond the initial 4 week course. We continue to explore options for coverage.

## Discussion

Granulomatous skin diseases can be challenging to treat. Although various therapeutic modalities have been described in the literature,^4^ there is a lack of definitive data demonstrating efficacy, highlighting the need for alternative, more efficacious yet safe treatment options. In our report of three patients, the oral JAK inhibitor tofacitinib resulted in significant improvement of ulcerative NL and long-standing refractory GA.

The mechanism of action of JAK inhibitors for treatment of granulomatous diseases is not well understood. A limited understanding of the etiopathogenesis of GA overall has largely impeded the development of molecularly targeted therapeutic options.^13^ Current understanding is that GA is a delayed-type T-helper 1 (Th1) cell-mediated hypersensitivity reaction to an unknown antigen; however, upregulation of Th2 and JAK signalling has also been identified.^18,19^ Th1 cells produce interferon-γ, a key cytokine that plays a role in macrophage activation and granuloma formation.^20^ Interferon-γ along with oncostatin M, interleukin (IL)-21, and IL-15, are upregulated in GA and are mediated by the JAK-STAT pathway.^13,19^ Therefore, it is likely that inhibition of these key cytokines via blockade of the JAK-STAT pathway may represent a targeted treatment approach for GA and other granulomatous diseases and explain the rapid effects of JAK inhibitor therapies.

To date, a limited number of publications have reported the usefulness of targeting the JAK-STAT pathway in granulomatous conditions. In two recent single-case reports, treatment of GA with 2% topical tofacitinib BID resulted in near complete resolution after 12–15 weeks.^10,11^ A recent prospective study showed that oral tofacitinib 5 mg BID induced complete or near-complete clinical remission in patients with recalcitrant GA (n = 1) and cutaneous sarcoidosis (n = 3), with clinical improvement noted as early as 8 weeks, and remission after 6–10 months.^12^ Similarly, oral tofacitinib resulted in clinical and histological remission of GA in three patients and significant improvement in the other two patients after 6 months.^13^ Several other reports have documented successful treatment of sarcoidosis with JAK inhibitors.^14–16^ Currently, a clinical trial of tofacitinib for cutaneous sarcoidosis and GA is underway (NCT03910543). We have found one case report of ulcerative NL responding to the JAK inhibitor, ruxolitinib, normally indicated for polycythemia vera.^17^

The rapid improvement noted in our three patients provides further encouraging evidence that JAK inhibitors may be a beneficial therapeutic option for granulomatous diseases including ulcerative NL and recalcitrant generalized GA. Limitations of our report is the relatively short treatment period, ranging from 1 to 10 months, but we felt it beneficial to share our observations with other clinicians who may be looking for new treatment options for patients with recalcitrant granulomatous diseases. Larger controlled trials for longer periods of time are needed to ascertain the long-term efficacy and safety of JAK inhibitors for granulomatous diseases.
